# Reviewing ecological implications of mycorrhizal fungal interactions in the Brassicaceae

**DOI:** 10.3389/fpls.2023.1269815

**Published:** 2023-11-22

**Authors:** Adam N. Trautwig, Michelle R. Jackson, Stephanie N. Kivlin, Kristina A. Stinson

**Affiliations:** ^1^ Stinson Laboratory, Department of Environmental Conservation, University of Massachusetts, Amherst, MA, United States; ^2^ Rocky Mountain Biological Laboratory, Crested Butte, CO, United States; ^3^ Kivlin Laboratory, Department of Ecology and Evolutionary Biology, University of Tennessee, Knoxville, TN, United States

**Keywords:** arbuscular mycorrhizal fungi, Brassicaceae, facultative mycorrhizal association, intraspecific variation, species-site interaction

## Abstract

Mycorrhizal associations are plant-fungal mutualisms that are fairly ubiquitous and likely evolved multiple times in phylogenic history; however, some plant families have consistently been identified as non-mycorrhizal, including the Brassicaceae. In this paper, we reviewed the literature and DNA databases for potential mechanisms that preclude mycorrhizal symbioses in the Brassicaceae and for exceptions to the general observation of non-mycorrhizal status within this plant family. In instances of association between members of the Brassicaceae and arbuscular mycorrhizal fungi we posed hypotheses for why these interactions occur in the species and sites observed. Instances of inconsistent association with mycorrhizal fungi were attributed to inter- and intraspecific variations in plant biology, disagreements in vernacular, methodology contradicting historical mycorrhizal surveys, and association being a facultative, variable trait that is determined by species-site interactions. We propose further research on a) the extent of mycorrhizal association in the Brassicaceae, b) the molecular mechanisms dictating association, and c) whether Brassicaceae-mycorrhizal fungal interactions result in nutrient transfer, and their particular roles in the family’s distribution across heterogeneous and harsh environments.

## 1 Introduction

The Brassicaceae (previously Cruciferae) is a monophyletic group composed of over 3,600 species and occupying a wide range of ecological roles across cosmopolitan and extreme environments (Al-Shehbaz et al., 2006; Al-Shehbaz, 2012; Anjum et al., 2012). It has been difficult to make meaningful generalizations about this family due in part to the range of environments they inhabit, but also due to the functional diversity present overall. Several whole genome duplication events are likely responsible for the rapid adaptive radiation and resulting broad level of diversification present across the Brassicaceae (Schranz et al., 2006; Al-Shehbaz, 2011; Franzke et al., 2011). However, the Brassicaceae is generally accepted to be non-mycorrhizal, due in part to the previous historical classification by DeMars and Boerner (1996) as well as to some notable examples of mycorrhizal suppression and/or non-mycorrhizal status within the family (Stinson et al., 2006; Veiga et al., 2013; Cosme et al., 2018). At the same time, the literature contains exceptions to this general observation, in which Brassicaceous species are known to form below-ground symbionts including with mycorrhizal fungi (Regvar et al., 2003; Pongrac et al., 2008; Almario et al., 2017; Fernández et al., 2019). Here we summarize the historical classification of the Brassicaceae as non-mycorrhizal and provide examples of mycorrhizal interactions with specific members of the Brassicaceae. We discuss some of the ecological and evolutionary considerations that might contribute to this discrepancy and review existing phylogenies including species that demonstrate ability to form mycorrhizal symbioses.

## 2 Classification of The Brassicaceae as non-mycorrhizal

Foundational investigations into the classification of certain groups of plants as non-mycorrhizal, particularly the Brassicaceae, outline the myriad reasons that may be responsible for this status (Tester et al., 1987). We found that over the intervening time these observations have consolidated into two overarching hypotheses, both largely focused on the agriculturally imperative *Brassica* genus. The first states that the absence of a mycorrhizal growth stimulator in the roots is likely responsible for the lack of mycorrhizal symbiosis (Glenn et al., 1988). The second asserts that antifungal compounds produced in the roots, potentially as an anti-pathogen defense, of some non-mycorrhizal plants may play a role in their non-mycorrhizal status (e.g., Anthony et al., 2020).

### 2.1 Lack of mycorrhizal growth stimulator

Recent focus has determined molecular mechanisms, or lack thereof, responsible for the observations that numerous members of the Brassicaceae either do not associate with mycorrhizal fungi and form functional arbuscules (**Box 1**; DeMars and Boerner, 1996) or form associations that produce a measurable reduction in growth when associations do occur (Veiga et al., 2013). Delaux et al. (2014) identified a group of “symbiosis specific genes” that members of the Brassicaceae lacked. Specific to the order Brassicales 11 genes are depicted in Figure 1 of Delaux et al. (2014): NFP, DMI2, CASTOR, DMI3, IPD3, RAM1, RAM2, VAPYRIN, STR, STR2, and PT4. These genes code for proteins with a variety of functions including perception, communication, and development of mycorrhizal symbiosis but also functions which may indirectly affect mycorrhizal association (i.e. calcium spiking, transcription factor, and several proteins with unknown functions) (Delaux et al., 2013). These genes and associated transcripts are highly conserved in land plants and their absence was confirmed in a phylogenetically diverse group of members of the Brassicaceae (Delaux et al., 2014). Additional searches for genes linked to arbuscular mycorrhizal symbiosis has yielded interesting results with predicted symbiotic functions (e.g. ion channel proteins, transmembrane proteins, and protein kinases) (Bravo et al., 2016), some of which have orthologs in the Brassicaceae (e.g. the CCD protein family and CBX1) (Almario et al., 2022). The functions of these proteins must be definitively ascertained before causation can be ascribed. An analysis outlined in Sharma et al. (2022) utilizing OrthoFinder (Emms and Kelly, 2015) found that of 14 AM symbiosis-specific genes nine putative orthologs were identified in proteome data of four members of the Brassicaceae. Similarly, of 11 common symbiotic pathway genes six putative orthologs were found, leading the authors to conclude that the absence of common symbiotic pathway genes may not be the sole reason for a lack of AM fungal association with the Brassicaceae (Sharma et al., 2022).

Box 1 **Arabidopsis thaliana.**

*Arabidopsis thaliana* (L.) Heynh has long been considered a model organism for research in plant biology and has been an especially useful molecular resource since its whole genome sequence was published in 2000 (Meinke et al., 1998; Arabidopsis Genome Initiative, 2000). The focus on *A. thaliana*, relative to other members of the Brassicaceae, is also likely one of the strongest reasons for the enduring classification of the Brassicaceae as a non-mycorrhizal family. Vesicular arbuscular mycorrhizal development is considered typical when hyphae, vesicles, and arbuscules are present (DeMars and Boerner, 1996). Vesicles are broadly considered storage structures, while arbuscules are generally responsible for the association with a plant partner (Smith and Read, 2008) with nutrient exchange, specifically translocation of P from fungal hyphae to internal plant structure occurring through arbuscules (Smith et al., 2015).

Although AM fungi colonize *A. thaliana* roots, arbuscules do not form. In those cases, when forced to interact with mycorrhizal fungi, as shown with *A. thaliana* in Veiga et al. (2013), there can be a 50% decrease in plant growth. Many mechanisms can explain the lack of functional mycorrhizal association. Key among them is the presence of a complex spatial arrangement of microbe associated molecular pattern (MAMP) response mechanisms in *A. thaliana* roots, as well as the absence of several nutrient transporters specific to mycorrhizal symbiosis (including PT4) (Delaux et al., 2014). MAMP responses are complex, with response to flagella associated polypeptide from *Pseudomonas aeruginosa* and peptidoglycan in the elongation zone and chitin in more mature root tissue (Millet et al., 2010). Similarly, *A. thaliana* has MAMP triggered immunity to potentially beneficial interactions with *Rhizobium*-legume forming symbionts that can be mitigated through the application of Nod factor (Liang et al., 2013). *Arabidopsis thaliana* does have its own beneficial root associates with a core microbiome being well investigated (Lundberg et al., 2012; Zolla et al., 2013; Bai et al., 2015). One interaction involves *Colletotrichum tofieldiae*, which translocates plant available phosphate to its host via root associated hyphae only under phosphate starvation conditions. The closely related pathogen *C. incanum* does not perform this function (Hiruma et al., 2016). Additionally, *Serendipita* [formerly *Piriformospora*] *indica*, an endophytic fungus that colonizes the roots of *A. thaliana* and benefits its plant partner through a complex and highly regulated cooperation (Peškan-Berghöfer et al., 2004; Camehl et al., 2010). This interaction has been shown to modulate both nutrient concentration in plant roots and plant defense, resulting in increased growth of the plant partner (Opitz et al., 2021). This interaction may provide a roadmap to better understanding the function of mycorrhizal interactions with members of the Brassicaceae as well as fungal perception by would be plant partners.

### 2.2 Antifungal compounds: glucosinolates

Production of specialized chemical defenses in the Brassicaceae may produce an atypical rhizosphere system that could play a role in plant-plant and plant-microbe interactions. Of particular note are the glucosinolates; volatile and semi-volatile compounds that result from glucosinolate degradation, and hydrolytic products of glucosinolates—isothiocyanates (Ahuja et al., 2010; Schranz et al., 2011; Brolsma, 2014). The role of glucosinolates in non-mycorrhizal status has been enumerated by Tester et al. (1987).

Glucosinolate degradation occurs when fresh parts of the plant are crushed, releasing myrosinase, an enzyme responsible for hydrolysis of glucosinolates into isothiocyantes (Al-Shehbaz, 2011). In addition to their properties associated with the Brassicaceae, numerous researchers have demonstrated isothiocyanates can also have anti-microbial effects which protect against herbivory as well as pathogen infection (Bennett and Wallsgrove, 1994; Fan et al., 2011), but may suppress root symbioses with soil microbiota (Walker et al., 1937; Klöpping and van der Kerk, 1951; Bradsher et al., 1958; Wolfe et al., 2008; Plaszkó et al., 2021). In particular, the widely invasive *Alliaria petiolata*, garlic mustard, can outcompete native AM-fungal dependent plants by suppressing spore germination and root colonization by AMF on native plant species (**Box 2**; Roberts and Anderson, 2001; Stinson et al., 2006; Roche et al., 2020). Recent studies have definitively demonstrated the presence of orthologous proteins linked to indolic glucosinolates in phylogenetically diverse groups in the Brassicaceae. These proteins also experimentally reduce AMF colonization in *A. thaliana* (Anthony et al., 2020).

Box 2 **Alliaria petiolata.**

Another well examined interaction between a member of the Brassicaceae and mycorrhizal fungi involves the well-characterized invasive species *Alliaria petiolata—*a biennial, non-mycorrhizal, herbaceous plant, originating from Eurasia, that has proliferated in woodlands in the eastern United States and Canada (Nuzzo, 1999; Stinson et al., 2006). *Alliaria petiolata* produces glucosinolates that are novel in North America, as well as other secondary metabolites, that negatively affect growth of native plant neighbors in its introduced range by suppressing mycorrhizal fungi (Callaway et al., 2008; Barto et al., 2010). These compounds have been implicated in the suppression of mycorrhizal fungal inoculum potential that is not found in the native range of *A. petiolata* (Roberts and Anderson, 2001). Lower levels of colonization by AM fungi on *Acer saccharum, Acer rubrum*, and *Fraxinus Americana* were observed in soils that had been invaded by *A. petiolata* (Stinson et al., 2006). Reductions in ectomycorrhizal fungi were observed in a separate multi-pronged experiment, which included EM fungal root tip biomass and EM fungal colonization (Wolfe et al., 2008). Additionally, Anthony et al. (2017) and Duchesneau et al. (2020) both determined that novel saprobes and pathogens were found in invaded versus uninvaded patches, along with numerous shifts in broad functional groups, and an overall increase in fungal richness associated with *A. petiolata* invasion. These shifts in belowground communities persist for over a decade following *A. petiolata* invasion resulting in a cascade of effects on plant physiology and resource use (Bialic-Murphy et al., 2021) and shifts in native plant community composition (Roche et al., 2020).

### 2.3 An ecological perspective to non-mycorrhizal status

Given that molecular mechanisms linked to loss of ability in members of the Brassicaceae to associate with mycorrhizal fungi have been identified (Delaux et al., 2014; Domka et al., 2019), we decided to focus our review on where mycorrhizal association have been observed and the related ecology of those organisms. Specific questions we developed related to the extent, and nature, of mycorrhizal association with species within Brassicaceae included:

- Is there an impact of mycorrhizal fungi on the plant?
- Does the plant serve as habitat for mycorrhizal fungi?
- Is there a landscape or population level implication to this interaction?

We assessed the frequency of AM fungal colonization of Brassicaceae species by conducting a literature review of microscopic investigations of Brassicaceous roots and a data-mining exploration of AM fungal DNA from surveys of Brassicaceae roots. Both surveys found some presence of AM fungal structures and DNA throughout plants in the Brassicaceae. Here we discuss these findings in more depth and provide suggestions for future directions in the field.

## 3 Literature review of exceptions to non-mycorrhizal status in the Brassicaceae

**Figure 1:**
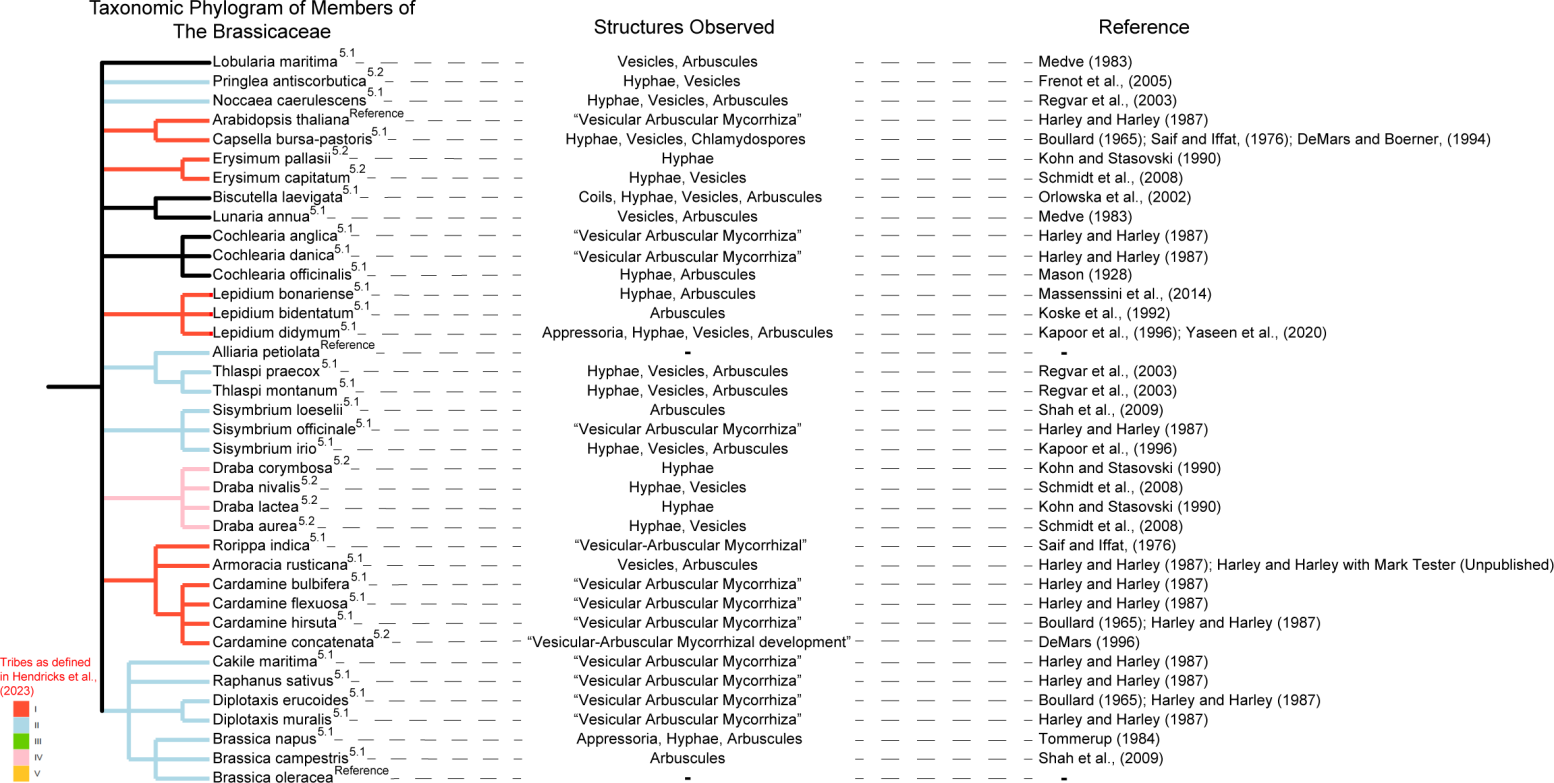
Several examples are present in the literature of association between members of the Brassicaceae and mycorrhizal fungi. Species exhibiting these associations have been grouped according to their taxonomic relatedness (following the NCBI taxonomic database) in a dendrogram, with annotation of Brassicaceae Tribe (following Hendriks et al., 2023) denoted by color of phylogram branch. Branches without color were classified by Hendriks et al. (2023) as “rouge taxa” which resisted classification into one of the 5 tribes. Location in the text is represented by superscript of section number. Specific structures observed are listed by cited article, with *Arabidopsis thaliana*, *Alliaria petiolata*, and *Brassica oleracea* included for reference.

In a comprehensive review of the literature searching the terms “Mycorrhizal Fungi/al”, “Association”, “Arbuscule”, and “Brassicaceae/Cruciferae” in Web of Science we found examples of arbuscule formation in numerous phylogenetically and ecologically disparate Brassicaceae species through either search results or relevant articles in referenced articles (**Figure 1**). Below we summarize the nature of these associations and pose some hypothetical ecological explanations.

### 3.1 Mycorrhizal associations detailed in published literature

#### 3.1.1 *Armoracia rusticana* Gaertn., May. & Scherb.

Harley and Harley (1987), in addition to serving as a reference list of mycorrhizal association presents a few novel observations. Two are relevant to this list, *Armoracia rusticana* was collected by Harley and Harley (and examined in cooperation with Mark Tester) and is described as having “[m]any vesicles, occasional arbuscules”. Similarly, these authors describe *Iberis amara* as being moderately colonized, although no mention of specific structures is detailed. No further mentions of sites or descriptions is present but, along with *Arabidopsis thaliana*, *Biscutella laevigata* (section 5.4), *Brassica napus* (section 5.3), *Brassica oleracea, Cakile maritima, Capsella bursa-pastoris* (section 5.5), *Cardamine bulbifera*, *Cardamine flexuosa*, *Cardamine hirsuta*, *Cochlearia anglica*, *Cochlearia danica*, *Cochlearia officinalis* (section 5.6), *Diplotaxis erucoides*, *Diplotaxis muralis*, *Raphanus sativus*, and *Sisymbrium officinale*, these plants are characterized by Harley and Harley (1987) as “Vesicular-arbuscular (VA) mycorrhiza”. Where possible, these instances were traced back to their original source. Several records did not meet our standard for inclusion in this list. In other cases, the original text was unable to be located or is in Polish.

#### 3.1.2 Brassica campestris L. and Sisymbrium loeselii L.

A survey of invasive plants located in the Kashmir Valley of the Himalaya found two members of the Brassicaceae that hosted arbuscule forming mycorrhizal fungi. The authors noted that while most species survey presented as either *Arum*-type or *Paris*-type both *B. campestris* and *S. loeselii* were characterized as intermediate of those classifications (Shah et al., 2009).

#### 3.1.3 Brassica napus L.

Observation of arbuscule formation by AM fungi on *Brassica napus* L. was documented in Tommerup (1984). Conditions in which *B. napus* was grown included steamed sand with added nutrients in a growth cabinet. Levels of arbuscule formation were low particularly in comparison to *Trifolium subterraneum* and the functional significance of the associated structures present inside and outside of the root has been questioned by some (Tommerup, 1984; Brundrett and Tedersoo, 2018).

#### 3.1.4 Biscutella laevigata L.

Members of *B. laevigata* and *Plantago lanceolate* were surveyed for mycorrhizal colonization on mine waste mounds in Poland. Arbuscule formation was found during the flowering stage on contaminated zinc sites and on noncontaminated sites in the Tatra Mountains. Unique to other members of the Brassicaceae the authors note the presence of vesicles, coils, and arbuscules (Orłowska et al., 2002).

#### 3.1.5 Capsella bursa-pastoris (L.) Medik. and Rorippa indica (L.) Hiern.

In northern Pakistan a survey of several plant families was conducted in order to evaluate mycorrhizal association across a variety of habitats. Among the plants shown to associate with mycorrhizal fungi were *C. bursa-pastoris* and *Rorippa indica*, which the authors described as having medium and low AMF association, respectively (Saif and Iffat, 1976). In addition, Boullard (1965) as relayed by Harley and Harley (1987) described *C. bursa-pastoris* as “exceptionally [Vesicular-arbuscular]”. These were the only members on this list in which observation of arbuscules was not explicitly noted. However, the characterization of the “VA mycorrhizal infection” as medium with regards to *C. bursa-pastoris* (Saif and Iffat, 1976), as well as the observation that members of *Capsella* have been observed to be devoid of indole glucosinolates (Bednarek et al., 2011; Hiruma et al., 2018) which some researchers have demonstrated plays a role in excluding mycorrhizal fungi in *A. thaliana* (Anthony et al., 2020), warranted its inclusion on this list. *Capsella bursa-pastoris* has also been observed to form internal hyphae, vesicles, extramatrical hyphae and chlamydospores but not arbuscules in three of four sites sampled in Ohio (DeMars and Boerner, 1994) and not displaying mycorrhizal colonization in Pennsylvania and California (Medve, 1983). In Saif and Iffat (1976) *Matthiola flavida* Boiss. and *Nasturtium officinale* W.T. Aiton were not observed to be mycorrhizal.

#### 3.1.6 Cochlearia officinalis L.

In an investigation of mycorrhiza in the roots of salt marsh plants in two locations in Wales, eight plants including the member of the Brassicaceae *Cochlearia officinalis* had fine rootlets examined for mycorrhizal presence and association. These plants specifically were noted as having hyphae in the cortical cells which in most cases was branched and non-septate (characteristic of AM fungi). In addition to branched, non-septate hyphae, arbuscules were present, with higher levels of arbuscules observed in three of the non brassicaceous plants, relative to *C. officinalis*. The mycelium is noted as being highly abundant in all halophytes with no presence in the “old roots, rhizomes, aerial stems and leaves” (Mason, 1928). This last observation would seem to counter the assertion in DeMars and Boerner (1996) that these instances of mycorrhizal structures are parasitism of senescing roots.

#### 3.1.7 Lepidium bonariense L.

In a survey of weed species present in agricultural areas Massenssini et al. (2014) *Lepidium bonariense* was found to be colonized at relatively high densities with an *Arum*-type development. *Arum*-type developments are characterized by linear hyphae and arbuscules. The authors characterized the association as active and extensive although they did not denote the density of arbuscules specifically.

#### 3.1.8 Lepidium bidentatum var. o-waihiense (Cham. & Schlechtend.) Fosb.

Another example of variable association with mycorrhizal fungi was enumerated by Koske et al. (1992) in which an endemic Hawaiian member of the Brassicaceae *Lepidium bidentatum* var. *o-waihiense* was observed to form arbuscules. This observation was extremely limited with only 2 individuals being examined for associations. However, given the high degree of isolation in which the species is found the observation represents a valuable data point.

#### 3.1.9 Lepidium didymum L. and Sisymbrium irio L.

Yaseen et al. (2020) investigated weed species in a wheat field in Tehsil Tangi of the district Charsadda in Pakistan. Plants were excavated with rhizospheric soil and washed before assessing 50 root segments for colonization and rhizospheric soil for spore density. Three members of the Brassicaceae were included in this survey: *Brassica nigra*, *Camelina sativa*, and [*Lepidium didymum*]. Rhizosphere relative spore density for *Glomus*, *Acalospora*, and *Sclericystis* was 49.33, 3.33, and 0.67% of all spores for *B.* nigra, 35.67, 9.67, and 0.67% for *C. sativa*, and 29.33, 2.33, and 1.33% for [*L. didymum*]. Mycorrhizal structures: external hyphae, internal hyphae, vesicles, and arbuscules in percent of segments surveyed corresponded to 0, 0, 0, and 0% in *B. nigra*, 0, 0, 0, and 0% for *C. sativa* and 1, 0, 6, and 15% for [*L. didymum*]. The authors note that, generally, colonization increased with plant age.

*Lepidium didymum* (syn: *Coronopus didymus*) and *Sisymbrium irio* were collected from the botanical garden of the University of Delhi and assessed at 15-day intervals for mycorrhizal association. In *L. didymum* numerous mycorrhizal structures were observed including appressoria, “S”-shaped and coiled internal hyphae, vesicles, and arbuscules that filled most of the cells. *Sisymbrium irio*, however, showed widespread hyphae, and vesicles but only a few arbuscules in “1-2 cells of the host plant” (Kapoor et al., 1996).

#### 3.1.10 Lobularia maritima (L.) Desv. and Lunaria annua L.

In a survey conducted in both Pennsylvania and California three replicates each of 25 members of the Brassicaceae were collected from the field and observed microscopically. *Lobularia maritima* and *Lunaria annua* were found to be mycorrhizal in this survey. The association was characterized by arbuscules which the authors described as sparse and restricted to the innermost layer of the cortical parenchyma (Medve, 1983). The authors also mention the presence of vesicles which they describe as sparse in *L. maritima* but not in *L. annua.*

#### 3.1.11 Thlaspi praecox Wulf., Noccaea caerulescens J. & C. Presl, and Thlaspi montanum L.

A variety of habitats were surveyed by Regvar et al. (2003) across Austria, Germany, Italy, and Slovenia. Meadow species of *Thlaspi* and *Noccaea* varied in presence or absence of arbuscule formation and, in the case of presence, abundance between individual and site. The arbuscules found to form in *T. praecox*, *N. caerulescens*, and *T. montanum* were from the *Glomus intraradices* complex and, interestingly, showed variation between *Thlaspi* spp. individuals. Further, although the sequences of *G. intraradices* nested within *G. intraradices* in phylogenetic analysis they diverged significantly from each other as well as from databank sequences (Regvar et al., 2003). This phylogenetic divergence would seem to suggest that members of the Brassicaceae may play a role in hosting unique populations of mycorrhizal fungi.

### 3.2 Unclear cases lacking some structures necessary to form “functional symbioses”

Numerous other species in the Brassicaceae have also been surveyed for mycorrhizal fungal colonization and found not to form associations with AM fungi. In examining the record of mycorrhizal associations that appear in the Brassicaceae we referred to Cosme et al. (2018) and Soudzilovskaia et al. (2020) for definitions and nomenclature. Arbuscular mycorrhizal associations require the presence of arbuscules or coils (Brundrett, 2009; Brundrett and Tedersoo, 2018). Given the recent supposition in Bueno et al. (2019), however, that arbuscules may not be required for a functional symbiosis and that other fungal structures, and vesicles in particular, may be indicative of an association we also included cases in this review in which details were incomplete, or several other fungal structures were present. Finally, we consider, given the abundance of diverging cases of mycorrhizal association outlined above, the possibility that some species in the Brassicaceae may be facultatively mycorrhizal plants developing associations in some conditions, but remaining non-mycorrhizal in other conditions (Brundrett, 2017).

Often there are instances of AM fungi being found in the roots of non-mycorrhizal host plants without the arbuscule formation necessary for a functional symbiosis. In a survey conducted in the field, one group dismissed potential evidence of mycorrhizal association within the Brassicaceae, seemingly for no other reason than its traditionally non-mycorrhizal status despite “…irregular,… hyphal penetration in roots of [*Draba*] *lactea*, *D. corymbosa*, and [*Erysimum*] *pallasii*…” which they referred to as “presumably non-mycorrhizal” without any further explanation (Kohn and Stasovski, 1990). *Pringlea antiscorbutica* from a sub-Antarctic island in a separate study was found to associate with AM fungi without forming arbuscules (Frenot et al., 2005). Several species including *Draba aurea*, *D. nivalis*, and *E.* [*capitatum*] were found on the Front Range of Colorado with some AM fungal hyphae and vesicles, but again lacking arbuscules (Schmidt et al., 2008). In Boullard (1965), as relayed by Harley and Harley (1987), *Caspsella bursa-pastoris*, *Cardamine hirsuta*, and *Diplotaxis erucoides* are described as being “exceptionally [vesicular arbuscular], unfortunately we were unable to examine Boullard (1965). The most thorough survey to our knowledge found that of 646 species examined in the greenhouse 18.9% showed some degree of association with arbuscular mycorrhizal fungi although none formed functioning arbuscules (DeMars and Boerner, 1996). The authors theorize that the lack of functional arbuscules may indicate that these are not associations but rather AM fungi parasitizing senescing roots. This would seem to be supported by the disagreements, within this same paper, between observations in the greenhouse and citations of earlier literature. DeMars (1996) makes a similar assertion when postulating the reason for the mycorrhizal fungi found in association with *Cardamine concatenata* at one site in one year but not in other sites or in other years.

## 4 The role of nurse plants and reciprocal reward in mycorrhizal interactions with the Brassicaceae

One of the wider sources of variation in mycorrhizal association with members of the Brassicaceae is between field observations and experimental or greenhouse cultures. Ocampo et al. (1980) is an early example that describes the nurse plant phenomenon, whereby cabbage, kale, and rape while grown in isolation show no indication that mycorrhizal fungi are present in roots. However, when grown with a mycorrhizal host lettuce, potato, and barley respectively a weak presence of hyphae are present in the roots of the member of the Brassicaceae. In all instances outlined in Ocampo et al. (1980) none of the tell-tale signs of association (i.e. arbuscules) are present but vesicles are occasionally present with numerous hyphal entry points. An examination of bulk soil and rhizosphere soil of *B. napus* showed that even after 10 years of monoculture a persistent and diverse AM fungal community was present in the soil with several AM fungi [OTUs] in the *B. napus* rhizosphere (Floc’h et al., 2022). Another experimental scenario that demonstrates the efficacy of nurse plants in stimulating mycorrhizal presence in a member of the Brassicaceae involves *A. thaliana* grown with either *Trifolium pratense* or *Lolium multiflorum* (Veiga et al., 2013). Growth of *A. thaliana* was tangibly curtailed in this experiment (**Box 2**). Fernández et al. (2019) evaluated biomass and transcriptional signals in *A. thaliana* after 8 weeks of growth in a bicompartental microcosm experiment similar to Veiga et al. (2013), with *Medicago truncatula* as the nurse plant species. Root colonization of *A. thaliana* was much lower than in nurse plant *M. truncatula* with *A. thaliana* lacking arbuscules (such as were seen in *M. truncatula*) but with intraradical hyphae penetrating root cortex. Although strigolactone biosynthesis genes CCD7 and CCD8 were activated in *A. thaliana*, indicating detection, transcripts of well characterized defense genes (MYB51, CYP71A12, PRB1 and ERF4) were not activated. Our interpretation of these investigations is that AM fungi can be made to interact with members of the Brassicaceae, to the detriment, or with seemingly no effect on the plant host. These fungi can maintain their presence, in diverse assemblages, after a decade of monoculture growth. The assertion that these groups do not interact, or that only senescing roots are impacted does not appear to be as plausible as a broader range of interactions where plants are impacted by the presence of diverse fungal communities and fungal communities can use non-host plants in an otherwise inhospitable location.

Somewhat counter to the example of nurse plants is the reciprocal reward hypothesis which states that a marketplace of phosphorus and carbohydrates exists between fungal and plant participants with incentivized beneficial exchange leading to the exceptional evolutionary stability that has been observed (Kiers et al., 2011). This explanation does not leave room for the observed nurse plant phenomenon as parasitized roots would presumably be cut off if more productive roots are still available. Similarly, the rather limited view of mycorrhizal symbiosis as phosphorus for carbohydrates and vice-versa is an oversimplification ignoring distribution of potential plant partners and the myriad benefits of mycorrhizal association (Walder and Van Der Heijden, 2015).

## 5 Molecular evidence of AM fungal colonization in the Brassicaceae

While DNA sequencing alone does not provide definitive evidence of a functional symbiosis (Soudzilovskaia et al., 2020), several studies have sequenced AM fungal 18S genes from roots of species within Brassicaceae. A search of GenBank (updated 06/15/2020) revealed 89 AM fungal DNA sequences from five studies in Asia and Europe (**Supplemental Table 1**). AM fungal DNA from 41 virtual taxonomic units (Öpik et al., 2010) and three AM fungal families, was isolated from five plant species (*Arabis hirsuta*, *Cardamine bulbifera*, *Thlaspi arvense*, *T. caerulescens*, and *T. praecox*).

### 5.1 Phylogenetic analyses of the Brassicaceae

The evolutionary relationships between Brassicacaeous species have only become more resolved in the past few years. Extensive phylogenetic studies conducted by Favre et al. (2014); Huang et al. (2015); Bravo et al. (2016); Guo et al. (2017), and Edger et al. (2018), which are based on previous work by Al-Shehbaz et al. (2006); Franzke et al. (2011) and others, have elucidated important insights into this plant group; including as a series of six distinct clades (A-E) that organize the Brassicaceae. Furthermore, the improvements in these phylogenies compared to previous research is based on the greater accessibility of both genomic, transcriptomic, and proteomic data, as well as advances in sequencing techniques. However, to our knowledge, there is only one study to date that uses phylogenetics to investigate the evolution of mycorrhizal associations specifically with members of the Brassicaceae.

**Figure 2:**
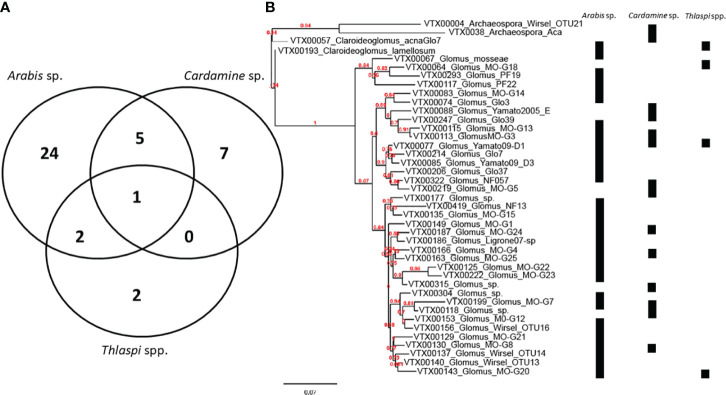
Virtual Taxa are shown by **(A)** specificity of associate in the Brassicaceae as well as **(B)** in a phylogram with associate indicated on the right-hand side. For phylogram, reference sequences were downloaded from MAARJAM database and phylogram was constructed using phylogeny.fr. Sequencing depth may have varied among the plant taxa studied.

A study conducted by Delaux et al. (2014) investigated the evolution of AM fungi for host genomes within several species of the Brassicaceae and found that the loss of genes associated with symbiosis may be responsible for the non-host status of many species in the Brassicales. This study by Delaux et al. (2014) is unique in that it sets the foundation for further investigation into these complex evolutionary relationships; but it does not include many of the species we have cited as forming mycorrhizal associations or arbuscules. As the genomic resources on which the analyses in Delaux et al. (2014) were based continue to develop, we expect to see more clarity into why a family in which mycorrhizal associations have been observed does not appear to have the genes necessary for symbiosis. An example of using these genomic resources to fully explore the permeation of mycorrhizal-Brassicaceae interactions includes continuing to identify instances of association (**Figure 2**). These reasons were broached in Cosme et al. (2018) as including which symbiotic genes are lost in non-host plants and conversely to Cosme et al. (2018), which genes remain in host plants, as well as where redundant mechanisms exist that support arbuscular mycorrhizal colonization. In addition, if these analyses are conducted by examining members of the Brassicaceae and accepted host lineages, and mycorrhizal association does occur in the Brassicaceae, research such as Favre et al. (2014) and Bravo et al. (2016) risk underestimating the molecular pathways involved in symbiosis despite otherwise sure methodological footing.

Of those species in Clade B (Huang et al., 2015; Guo et al., 2017), several members form mycorrhizal associations. All 3 *Draba* species represented and 2 of the *Thlaspi* species discussed earlier form mycorrhizal associations. These observations necessitate the question of whether certain genera are more likely to form mycorrhizal associations. This is likely a complex question as we observe associations for some species in the same genus (e.g., *Lepidium bonariense)* but not others (*Lepidium latifolium)* as well as an area of interest for future surveys seeking to better define the extent of association in the Brassicaceae.

Another important component to consider that we were not able to do here, is to compare the evolutionary rates of these species that make mycorrhizal associations. This would improve our comprehension as to how fast or slow certain genera may have lost the ability to form AM fungi as discussed in Delaux et al. (2014), in addition exploring species that associate with mycorrhizal fungi and/or the biases created from phylogenies due to limitations of available sequences.

## 6 Areas of future research

Several different definitions are used in the literature to denote the presence or absence of an AM fungal association. Most surveys we used, at a minimum, specified the structures present as hyphal, coils, vesicles, and arbuscules (Medve, 1983; Kapoor et al., 1996; Orłowska et al., 2002). Some surveys were more restrictive, either describing the mycorrhizal development as low, medium, high (Saif and Iffat, 1976) or only listing percent colonization (Akhmetzhanova et al., 2012). In addition, changing definitions of AM fungal associations are abundant with some researchers characterizing an association as rudimentary, when arbuscules are present in low concentration (Cosme et al., 2018), while other authors present these cases as non-functional and therefore non-mycorrhizal (Brundrett and Tedersoo, 2019; Sun et al., 2019). Likewise, consistency in presence and absence of mycorrhizal association between individual species is absent, requiring additional research on the extent of inter- and intraspecific variation (i.e. *Chorispora tenella* and *Descurania pinnata*) (Reeves et al., 1979; DeMars and Boerner, 1996). Investigation into the exchange of nutrients between AM fungi and plant hosts is necessary to clarify these inconsistencies.

We propose that rather than any individual mechanism being responsible for placement of a specific case along the parasitism/mutualism spectrum various mechanisms likely contribute to individual cases of AM fungal association with plants within the Brassicaceae, including the presence of certain genes/orthologs (Delaux et al., 2014; Cosme et al., 2018), glucosinolate profiles and production (Vale et al., 2015; Anthony et al., 2020), and site x species interactions (Reeves et al., 1979; DeMars and Boerner, 1996). A thorough survey of the Brassicaceae for mycorrhizal associations, including sampling species across numerous habitats and examining members that persist in extreme environments may help address several additional questions as we note below.

### 6.1 Intraspecific variations provide resistance to definitive classification of mycorrhizal status

As mentioned earlier, glucosinolates and products of glucosinolates produced through degradation, which are specific to members of the Brassicaceae, have anti-microbial effects (Walker et al., 1937; Klöpping and van der Kerk, 1951; Bradsher et al., 1958). Due to the influence these compounds have on fungal growth, the cycle of production within individual plants may be an important factor in determining the reason for variable reports of mycorrhizal association. In an examination of phenolic profiles, organic acid profiles, and antimicrobial activity of four different varieties of sprouts belonging to *B. oleraceae* (broccoli, Portuguese Galega, Portuguese Tronchuda cabbage, red cabbage) in different light treatments and over time, Vale et al. (2015) found that chemical profiles and antimicrobial activity varied among treatments. Even within different cultivars of the same crop, variations in root glucosinolate profiles occur among individuals within *B. oleracea* var. capitata (Kabouw et al., 2009).

In addition to variations in glucosinolate profiles and levels between species or cultivars, glucosinolates can also vary over time. Pongrac et al. (2008) found that in *Thlaspi praecox*, a mycorrhizal, heavy metal accumulating plant, the highest levels of glucosinolates were found in the roots during the vegetative, flowering induction, and senesce phase. In *T. praecox* peaks in mycorrhizal colonization frequency, global intensity, and intensity of colonized fragments peaked in the flowering phase (Pongrac et al., 2008). Meanwhile, concentrations of Cd, Zn, Pb, Fe also increase during flowering phase (Pongrac et al., 2007). This variation in phytochemical profile among species, among cultivars, and even among individuals over time may be an additional variable responsible for the inconsistencies in reports of colonization of members of the Brassicaceae by mycorrhizal fungi.

### 6.2 Elucidating the role of m-factors in the Brasscicaceae

Contrary to the ideas central to the hypotheses presented by Glenn et al. (1988) and Schreiner and Koide (1993) a study by Zeng et al. (2003) observed that far from just producing secondary metabolites with allelopathic affects, the Brassicaceae, especially *Brassica*, has members that produce compounds which act as “M-factors.” M-factor was originally used to describe root exudates from trees that when added to agar increased the rate of growth in usually slow growing ectomycorrhizal fungi in pure culture (Melin, 1954; Satyanarayana et al., 1996). In the Brassicaceae, the role of “M-factors” were hypothesized to be isothiocyanates, and “isothiocyanate related compounds,” that stimulated the growth of the mycorrhizal fungi *Paxillus involutus*. In addition, several species also stimulated growth in another mycorrhizal fungi *Pisolithus tinctorius* (Zeng et al., 2003). Members of the Brassicaceae produce a variety of other secondary metabolites as well—or compounds which have been observed not to be necessary for plant survival but enhance performance survival in the environment (Kliebenstein, 2004). Determining how these plant secondary metabolites affect colonization of mycorrhizal fungi across Brassicaceae species should be a goal for future research.

### 6.3 Mycorrhizal association can vary between site and over time

The investigation by Regvar et al. (2003) was conducted over a wide geographic area and is particularly valuable for determining if mycorrhizal association may be a variable trait due to the inconsistency in association found. In addition, the tendency of the Brassicaceae to form associations with mycorrhizal fungi, without forming mutualisms with those fungi may mean individual members of the Brassicaceae act as an “oasis” on a micro-spatial scale; providing a habitat without facilitating a relationship. The non-symbiotic association may be more ecologically significant in locations that are sparser and less hospitable to plant establishment and proliferation.

An example of extreme geographic isolation playing a role in the association between a member of the Brassicaceae and AM fungi may be evident in the Hawaiian endemic *L. bidentatum* var. o*-waihense* observed by Koske et al. (1992). The status of the Hawaiian Islands as the “most remote major archipelago on earth” justifies the questioning of whether similar associations may be found in similar conditions. An additional case of mycorrhizal association that may have arisen as a result of the occupation of an inhospitable environment was the colonization of *B. compestris* and *S. loeselii* in the Kashmir Himalaya (Shah et al., 2009). Surveys conducted in Soviet Russia have recently been made available that show an additional genus that consistently associates with mycorrhizal fungi. Across mountain, desert, arctic tundra, and taiga regions *Lunaria dolichoceras*, *L. peducellata*, and *L. vulgaris* showed strong to moderate colonization by mycorrhizal fungi (Akhmetzhanova et al., 2012). However, due to the age of the surveys, conducted from 1957–1975 and published sometime later, the extent of the association is unclear. Of these species found to associate with mycorrhizal fungi in the field only *S. loeselii* was examined by DeMars and Boerner (1996) and it was found not to associate with mycorrhizal fungi in the greenhouse.

The inconsistencies that are evident in the literature, in particular, between DeMars and Boerner (1996) and later field surveys indicate that just as association may vary among species over time, as levels of glucosinolates vary, it is also feasible that selection for interaction with mycorrhizal fungi may vary at the site level as well. This would result in a species forming mycorrhizal associations in one site but not another. More surveys of the same plant species across space and time are necessary to resolve how ecological contexts promote colonization of AM fungi on Brassicaceae species.

## 7 Conclusion

A thorough review of the literature shows an ecologically diverse group of members of the Brassicaceae that have structures present which are consistent with functional AM fungal association. This association was present across literature and DNA databases. There are some inconsistencies in research and terminology that might account for this discrepancy. The definition of AM fungal association has been interpreted in different research as including different structures and intensities. Recent research has proposed that vesicles could be indicative of a functional symbiosis or that members of the Brassicaceae may be facultatively mycorrhizal. The lack of toolkit genes has been put forth as a potential mechanism to explain the historic classification of the Brassicaceae as non-mycorrhizal. Additional genomics resources would be beneficial in identifying a molecular mechanism to cases of association in mycorrhizal plant species. A more comprehensive survey of this family for mycorrhizal association is necessary to answer remaining questions about the mechanisms and reasons for low AM fungal association in this family.

We formulated several research questions that may help to direct future avenues of investigation related to the role an individual species ecology plays in forming rudimentary or non-mycorrhizal associations. (1) How will climate change alter interactions between members of the Brassicaceae and AM fungi in novel environments as habitats shift upward in elevation and latitude? As climate change continues to introduce novel ecological interactions it is important to determine if any generalizations are available. (2) Are some invasive members of the Brassicaceae more damaging or pernicious on surrounding AM fungi than others and should there be priorities for treatment based on species or species x site interactions? In an increasingly globalized world, plant invasions will continue to strain limited resources allocated to land management and stewardship. While these remain only a few of the many open lines of inquiry into the prevalence, distribution, and function of AM fungi within the Brassicaceae family, investigating the impacts of global change on plant-mycorrhizal fungal interactions is critical for forecasting plant persistence and ecosystem function over the next century.

## Author contributions

AT: Conceptualization, Data curation, Investigation, Methodology, Project administration, Writing – original draft, Writing – review & editing. MJ: Investigation, Writing – review & editing. SK: Conceptualization, Data curation, Funding acquisition, Supervision, Visualization, Writing – original draft, Writing – review & editing. KS: Conceptualization, Funding acquisition, Resources, Supervision, Visualization, Writing – original draft, Writing – review & editing.
